# Regulatory role of the Cpx ESR in bacterial behaviours

**DOI:** 10.1080/21505594.2024.2404951

**Published:** 2024-09-18

**Authors:** Jiajia Wan, Xuejun Gao, Feng Liu

**Affiliations:** College of Animal Sciences, Yangtze University, Jingzhou, Hubei, China

**Keywords:** Envelope stress responses, Cpx response, virulence, antimicrobial resistance, inter-kingdom signaling

## Abstract

The envelope demarcates the boundary between bacterial cell and its environment, providing a place for bacteria to transport nutrients and excrete metabolic waste, while buffering external environmental stress. Envelope stress responses (ESRs) are important tools for bacteria to sense and repair envelope damage. In this review, we discussed evidence that indicates the important role of the Cpx ESR in pathogen-host interactions, including environmental stress sensing and responses, modulation of bacterial virulence, antimicrobial resistance, and inter-kingdom signaling.

## Introduction

Life forms, especially microbes, sense changes in their internal and external environments to regulate gene expression and metabolism. Bacterial pathogens protect themselves from external stress and toxic substances to preserve their structure and function within and between hosts and in polymicrobial communities. Bacterial pathogens sense their positions in relation to the host to effectively regulate the production of virulence factors. During these processes, the envelope of Gram-negative bacteria is the first structure that interacts with the host and the primary target for the host’s immune response against the bacterial pathogen.

The envelope of Gram-negative bacteria consists of an outer membrane (OM), an inner membrane (IM), and a periplasmic space (PP) containing a thin peptidoglycan (PG) layer [1]. The envelope delineates the boundary between the bacterial cell and its environment, providing a conduit for the bacteria to transport nutrients and expel metabolic waste. Additionally, it serves as a buffer against fluctuations in the external environment. Many environmental stresses and antimicrobials can cause envelope damage. Bacteria sense and repair envelope damage through the envelope stress responses (ESRs).

Several classic ESRs have been identified in *Escherichia coli* and other bacterial species, including the Cpx (conjugative pilus expression), σ^E^ (sigma factor), Bae (bacterial adaptive response), Rcs (regulator of capsule synthesis), and Psp (phage shock protein) systems [2]. Other potential ESRs, such as the envelope-spanning anti-sigma factors in *Bacteroides*, have also been identified [3]. The Cpx system is a classic two-component system (TCS) that responds to internal and external environmental pressures that may harm the envelope. Previous studies have shown that Cpx ESR plays a crucial role in adapting to environmental stimuli and regulating bacterial virulence [4,5], while recent studies indicate that it is also important in regulating antimicrobial resistance, and inter-kingdom signaling. To improve the understanding of the Cpx system, this paper will conduct a systematic review of its functions.

## Stress sensing and responses via the Cpx

**Figure 1.**
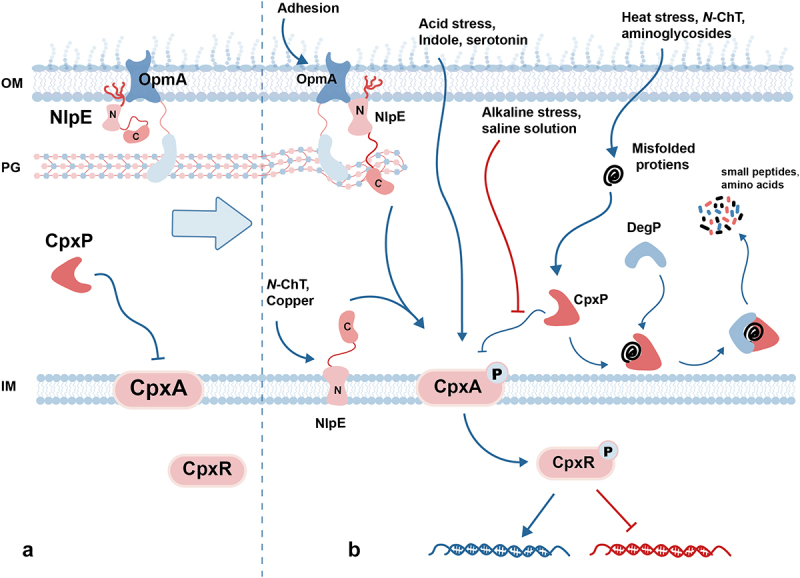
Signal transduction process of the Cpx ESR. (a) In the absence of the Cpx ESR induction signal, NlpE binds to OmpA to form a complex, and CpxP binds to CpxA to maintain CpxA in an unphosphorylated state. (b)The NlpE-OmpA complex senses adhesion signal and transmits it to activate CpxA through NlpE C-terminal domain. *N*-ChT and copper cause the retention of NlpE in IM, activating CpxA through the NlpE N-terminal domain. Acid stress, indole, and serotonin directly activate CpxA. Alkaline stress and saline solutions activate CpxA by inhibiting the binding of CpxP to CpxA. Heat stress, *N*-ChT, and aminoglycosides cause protein misfolding, which binds to CpxP and activates CpxA. DegP binds to a complex of misfolded proteins and CpxP, hydrolyzing them into small peptides and amino acids. The active CpxA autophosphorylates and transfers phosphate groups to CpxR, which regulates the expression of downstream genes.OM: outer membrane; IM: internal membrane; PG: peptidoglycan; *N*-ChT: *N*-chlorotaurine.

The Cpx system is comprised of the histidine kinase CpxA and the response regulator CpxR. Furthermore, the periplasmic protein CpxP and OM lipoprotein NlpE are essential components of the Cpx system for detecting and responding to stimuli, working in conjunction with CpxA and CpxR [5] (Figure 1a).

The activation mechanism of CpxA by NlpE has been the focus of debate among researchers. Whether the activation of CpxA is triggered by the C-terminal domain of NlpE, the impaired lipoprotein biogenesis or transport of NlpE, or the combination of both mechanisms concurrently activating CpxA, remains inconclusive [6]. Several recent studies indicated that NlpE may activate CpxA through both its N-terminal and C-terminal domain. In absence of the Cpx induction signal, the OM-anchored NlpE forms complex with OmpA through its N-terminal domain. In the presence of surface-related signals (such as surface adhesion or OmpA overexpression), NlpE and OmpA cooperate to transduce signals through the cell wall to CpxA via the NlpE C-terminal domain [7]. Impaired lipoprotein biogenesis or transport causes NlpE to get stuck in IM. In doing so, NlpE activates CpxA through the direct contact between its N-terminal domain (R73) and the CpxA periplasmic sensory domain (PSD) (D136, E138, D139) [8,9]. By inhibiting the acylation of NlpE, copper causes the OM-targeted NlpE to remain in the IM and activate CpxA [8]. Moreover, in the process of NlpE-dependent *N*-chlorotaurine (*N*-ChT) activating the Cpx in *Salmonella Typhimurium*, NlpE can not only sense the maturation defect of lipoprotein caused by *N*-ChT and directly activate CpxA through the N-terminal domain, but also sense the oxidative folding defect and indirectly activate CpxA through C-terminal disulfide bonds (Figure 1a) [10]. These results imply that the N-terminal activation and C-terminal activation mechanisms in NlpE are not mutually exclusive. NlpE can converge and transmit external signals to CpxA through different domains. In the N-terminal activation mechanism, the transition from an inhibitory signal to an active signal in CpxA is likely due to the binding advantage of NlpE. When NlpE accumulates in IM, there are more NlpE-binding sites (D136, E138, D139) than CpxP-binding sites (R35) in CpxA PSD, which allows NlpE to out-compete CpxP for binding to CpxA [9].

The Cpx ESR is crucial in monitoring and maintaining envelope homeostasis, and the role of Cpx depends on its capacity to detect and respond to internal and external environmental stimuli. Numerous studies have identified a wide range of stimulus signals that can activate the Cpx response (Figure 1b).

### Misfolded proteins

While the molecular mechanism underlying these signals remains unclear, the detection of misfolded proteins is thought to play a crucial role in sensing envelope stress signals. For example, the Cpx response to heat stress [11] (Figure 1b), copper toxicity detected by NlpE [8] (Figure 1b), and aberrant expression of Pap pili are all achieved by the detection of either directly or indirectly generated misfolded proteins [12,13]. Moreover, the Cpx ESR can also sense the endogenous toxic molecules generated by misfolding of periplasmic proteins, such as misfolded maltose-binding protein (MalE219) and misfolded Pap pilus subunit [14–18].

### Acid stress

While the response of Cpx to alkaline stress is well-known [5], its reaction to acid stress has only been documented in recent studies. Unlike the mechanism observed at alkaline pH, where the interaction between CpxP and CpxA is disrupted, acidification triggers the Cpx response directly by protonating histidine residues on CpxA [19] (Figure 1b). The Cpx response upregulates the *fabA* and *fabB* genes to increase unsaturated fatty acids, reduce membrane proton permeability, improve intracellular pH homeostasis, and facilitate bacterial survival in moderate acidic conditions [19]. *Acidithiobacillus thiooxidans*, an extreme acidophile, responds to acid stress by adjusting the composition of fatty acids in the cell membrane without an identified Cpx system, indicating the presence of Cpx-like system or mechanism [20–22]. Similarly, the ArsSR system in *Helicobacter pylori* contributes to acid stress resistance, resembling the Cpx system [20–22]. Furthermore, the Cpx response may induce the expression of acid tolerance response (ATR), thereby enhancing the growth of MCR-1-expressing bacteria in bile acid-containing media (moderate acidic conditions) [23]. Under extreme acidic stress, the Cpx response inhibits bacterial survival by repressing the expression of acid resistance (ARs) 2 [24]. ATR is used to resist moderate acid stress while ARs to adapt to extreme acidic conditions. The Cpx response facilitates the activation of ATR and represses that of AR2 to prevent erroneous induction, thus diminishing the fitness cost on bacteria. *H. pylori* and *Campylobacter jejuni*, extremophiles that reside primarily in acidic conditions, have been found to cope with acid stress through the ATR system, with no reported involvement of the Cpx system [25,26]. In addition to ATR, the Cpx response upregulates the expression of genes involved in cell wall modification and cross-talks with non-cognate response regulator OmpR [27]. OmpR functions as a global regulator of acid stress in S. *Typhimurium*, increases the replication and survival fitness of *Salmonella* during macrophage infection [28,29]. These mechanisms may collectively contribute to enhancing bacterial survival in moderate acidic conditions [19].

### Redox state

The Cpx response not only senses and coordinates acid stress adaptation but also serves as the primary ESR for maintaining envelope redox homeostasis. The Cpx response upregulates the transcription of *scsABCD* in *Salmonella* and *wecA* in *Actinobacillus pleuropneumoniae* to enhance bacterial resistance to exogenous oxidative stress [30,31]. It is worth noting that the Cpx response also has a protective effect against endogenous oxidative stress. By promoting disulfide bond formation, the Cpx response eliminates the cellular oxidative stress resulting from the inhibition of disulfide bond formation during long-chain fatty acids metabolism, thus preserving cellular redox balance [32]. The Cpx response also shields bacteria from oxidative stress induced by host immunity. When the host is infected by pathogens, immune cells produce reactive oxygen species (ROS) and reactive chlorine species (RCS) to eradicate the pathogens (see review [33]. *Neisseria gonorrhoeae*, which resides primarily within and in close proximity to neutrophils, induces the expression of redox factors through CpxAR homolog MisRS, such as bacterioferritin genes *bfrA* and *bfrB* [34], and transferrin binding protein genes *tbpB* and *tbpA* [35], adapting to oxidative stress environments. *N*-ChT, a kind of RCS, is mobilized by neutrophils to infection sites to eliminate *S. Typhimurium* [10,36]. The Cpx response senses *N*-ChT stress through NlpE (Figure 1b), induces the expression of periplasmic methionine sulfoxide reductase MsrP to repair *N*-ChT-oxidized proteins and combat *N*-ChT oxidative stress. Evasion of host immunity is a key factor for bacteria to sustain infection in the host. The Cpx response assists pathogens in evading host immunity, highlighting its crucial role in regulating bacterial virulence.

## Virulence modulation by the Cpx

The influence of Cpx ESR on bacterial virulence is one of the hot topics in the current research field, and a large number of virulence factors regulated by the Cpx response have been identified [37–44]. However, some interesting new studies highlight the novel role of Cpx in influencing bacterial virulence. The Cpx response regulates the expression of virulence factors through various mechanisms, including their spatio-temporal expression and coordination of energy metabolism. Additionally, the Cpx response indirectly impacts bacterial virulence by modulating the expression of adaptive genes [45–52].

### Spatio-temporal expression

**Figure 2.**
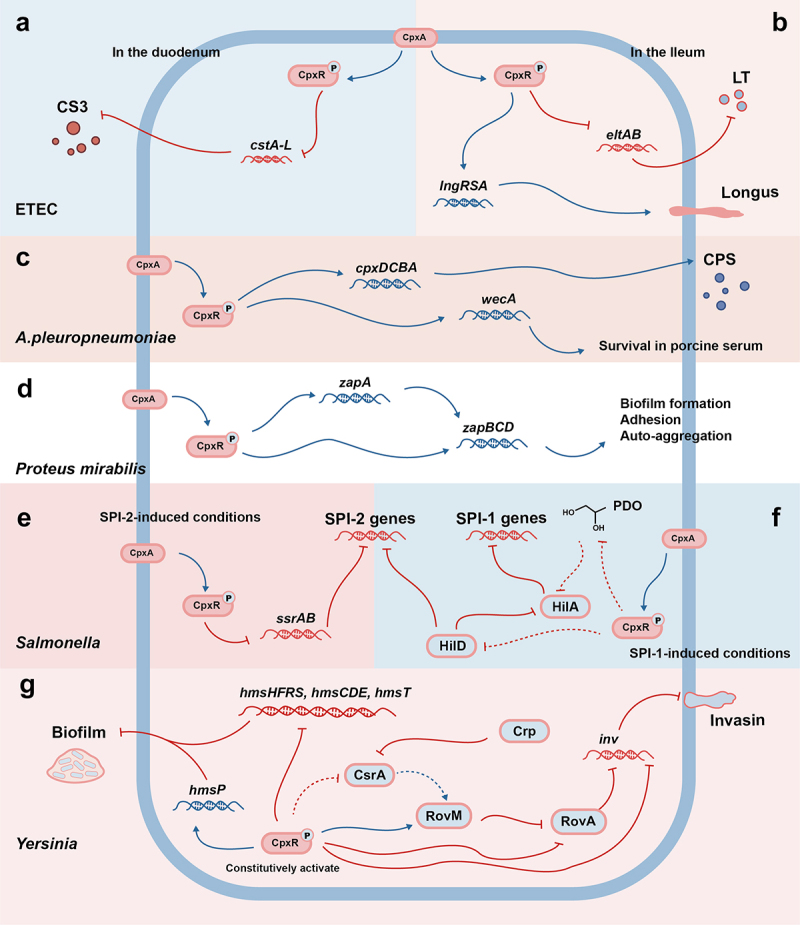
Virulence Modulation by the Cpx ESR. When EHEC is present in the duodenum, the Cpx ESR reduces the expression of CS3 by repressing the *cstA-H* gene cluster (a); When ETEC is in the ileum, the Cpx ESR reduces LT expression by inhibiting the *eltAB* operon and upregulates longus expression by promoting the transcription of the *lngRSB* operon (b). (c) The Cpx ESR in *A. pleuropneumoniae* upregulates CPS synthesis through the *cpxDCBA* operon and enhances the survival of porcine serum by upregulating *wecA* expression. (d) The Cpx ESR in *P. mirabilis* up-regulates *zapA* and the *zapBCD* operon, enhancing adhesion, auto-aggregation, and biofilm formation. When *Salmonella* in SPI-2-induced conditions, the Cpx ESR inhibits SPI-2 genes expression by binding to the *ssrAB* operon (e); When *Salmonella* in SPI-1-induced conditions, the Cpx ESR inhibits the expression of SPI-1 and SPI-2 genes by decreasing the stability of the SPI-1 Regulator HilD (f). (g) The Cpx ESR in *Yersinia* induces *hmsP* transcription but suppresses the transcription of *hmsHFRS*, *hmsT*, and *hmsCDE* thereby inhibiting biofilm formation. The Cpx ESR in *Yersinia* also inhibits the invasin expression through the Cpx-*inv*, Cpx-RovA-*inv*, and the Cpx-RovM-RovA-*inv* pathways. CS3: coli surface antigen 3; LT:Heat-labile enterotoxin; CPS: capsule polysaccharide; PDL: 1,2-propanediol.

Activation of the Cpx regulates the spatio-temporal expression of virulence factors in enterotoxigenic *Escherichia coli* (ETEC). The heat-labile (LT) and heat-stable (ST) enterotoxin, as well as type IV pilus Longus, are essential virulence factors of ETEC. Furthermore, the surface antigen 3 (CS3) serves as an important virulence factor of ETEC, playing a significant role in the colonization of ETEC in the small intestine [53]. The Cpx response suppresses LT expression by binding and blocking *eltAB* operon transcription under normal conditions [45] (Figure 2b). Under high glucose concentration and low bivalent cation levels, the Cpx response represses the expression of the *cstA-H* gene cluster [46], which encodes CS3, induces *lngRSA* expression, and enhances Longus synthesis [47]. Given the nutritional conditions in the ileum and duodenum, these studies support a model: The expression of Longus and LT occurs when ETEC reaches the duodenum, whereas CS3 and ST would be expressed when ETEC is located in the ileum [45–47] (Figure 2a,b). In this model, the Cpx response plays the essential function of distinguishing the ileum environment from the duodenum environment. Notably, this model results from multi-system coordination, where CS3 expression is regulated by multiple systems, and ST expression is not affected by the Cpx [46].

Similarly, the Cpx response in *Salmonella* coordinates the expression of virulence factors to minimize fitness costs. Firstly, from competing with other bacteria during invasion (*hilA*, *pocR*, *tatABC*) to evade the host immune system (*eco*, *ssrB*), the Cpx regulated genes play a crucial role in the early infection of *S. Typhimurium* [54]. Secondly, Effector proteins from *Salmonella* Pathogenicity Island 1 (SPI-1) and delivered by T3SS–1 are crucial for *Salmonella* to invade intestinal epithelial cells, while those from SPI-2 and delivered by T3SS–2 are essential for *Salmonella* to survive and replicate in host cells [55–57]. Under the condition of SPI-1 induction, the Cpx response inhibits the expression of SPI-1 and SPI-2 genes by decreasing the stability of the SPI-1 Regulator HilD, whereas under the condition of SPI-2 induction, the Cpx response inhibits SPI-2 gene expression by binding to *ssrAB* operon [48,49] (Figure 2e,f). This suggests that the Cpx response controls SPI-1 and SPI-2 expression through negative feedback, preventing excessive virulence factors expression and bacterial proliferation within macrophages.

### Coordination of energy metabolism

In *Salmonella*, HilD positively regulates HilA, while the Cpx response represses HilA through HilD [48,58–60]. Interestingly, HilA is suppressed by 1,2-propanediol (PDL), while PocR, a negative regulator of the *pdu-cob* cluster responsible for metabolizing PDL, is regulated by the Cpx [54,61,62] (Figure 2f). Although the mechanism by which PDL suppresses HilA and how the Cpx response coordinates with metabolism are still unclear, it still shows that the regulation of *Salmonella* virulence by the Cpx response is accompanied by the activation of metabolism and trade-offs in fitness costs.

Unlike the ambiguous correlation between metabolic activity and virulence factors expression in *Salmonella*, studies on *Yersinia pseudotuberculosis* show a clear link between internal nutritional conditions, external stress, and the expression of virulence factors. Previous research shows that the activated Cpx response can promote the expression of the pH 6 antigen chaperone-usher system, Ysc-Yop T3SS, invasin and other virulence factors by regulating the expression of the positive regulator RovA of *inv* (Cpx-RovA-*inv*) or directly regulating the *inv* operon (Cpx-*inv*) [50,63,64] (Figure 2g). New studies shows that the constitutively activated Cpx response regulates the transcription of RovA through RovM to repress *inv* expression in addition to the Cpx-RovA-*inv* pathway [65,66]. The up-regulated expression of RovM usually occurs in response to nutrition deficiency, suggesting that the Cpx response links internal nutrient restriction, external stress, and bacterial virulence via the CsrA-Crp-RovM-RovA-*inv* pathway, because this pathway is associated with the shift from acute to persistent infection in mice [65,67–70] (Figure 2g). The Cpx also influences the virulence of *Yersinia pseudotuberculosis* through the *hms* sites in addition to the pathways mentioned above. The constitutively activated Cpx response induces *hmsP* transcription but suppresses the transcription of *hmsHFRS*, *hmsT* and *hmsCDE* by binding to the *hms* promoter, thereby inhibiting biofilm formation [51] (Figure 2g).

### Other Modulation of Virulence

The Cpx response regulates virulence in various bacteria in addition to those mentioned above. In *A. pleuropneumoniae*, activation of the Cpx response affects capsule synthesis through the *cpxDCBA* operon, promoting survival in porcine serum by upregulating the expression of *wecA*, a gene encoding sugar-phosphate transferase WecA to initiate O-antigen repeating unit biosynthesis [31,71,72] (Figure 2c). The activated Cpx response up-regulates the expression of the *zapBCD* operon and ZapA protease in *Proteus mirabilis* to enhance adhesion, self-aggregation, and biofilm formation [73] (Figure 2d). The Cpx response also regulates the virulence of phytopathogens *Dickeya dadantii* 3937 and *Erwinia amylovora*. The Cpx response influences the virulence of *Dickeya dadantii* 3937 through the bis-(3'-5')-cyclic dimeric guanosine monophosphate (c-di-GMP) network, but its role in *Erwinia amylovora* is still unclear [74,75].

Not only the regulation of virulence genes, but also the ability to sense adhesion signals are crucial factors in assessing bacterial virulence. It has been suggested that the Cpx response senses adhesion through NlpE, but this hypothesis is highly debated (see review [6,76]). Recent studies suggest that the interaction between NlpE and OmpA may be the mechanism for the Cpx ESR to sense adhesion, but further research is needed for validation [7] (Figure 1b).

### Adaptive genes expression

Unlike the direct regulatory mechanism mentioned above, an intriguing study proposes that the virulence decrease caused by the Cpx response deficiency in *Citrobacter rodentium* may be a cumulative effect of Cpx-regulated genes on fitness. In this research, the individual deletion of Cpx-regulated genes does not affect the virulence phenotypes, but deleting CpxAR reduces the virulence, possibly due to the collective effect of Cpx-regulated genes on bacterial growth [52]. Similar findings have been reported in previous studies. In the *cpxRA* deletion strain, the *in* *vivo* virulence of *C. rodentium* decreased, independent of an altered growth rate or a defective type III secretion system [77]. Additionally, while the Cpx-regulated genes *degP* and *dsbA* are essential for *C. rodentium* infection, they do not fully account for the attenuation of *in vivo* virulence [78].

## Antimicrobial resistance mediated by the Cpx

**Figure 3.**
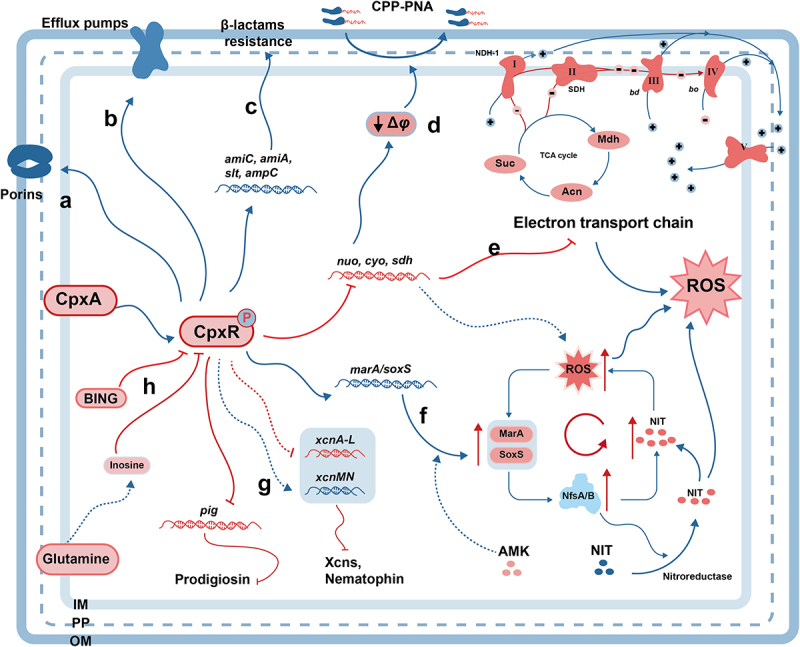
Antimicrobial Resistance Mediated by the Cpx ESR. The Cpx ESR upregulates porins (a), efflux pumps (b), and the expression of *aimC*, *amiA*, *slt*, and *ampC* (c) to confer bacteria resistance to antimicrobial agents. (d) The Cpx represses the expression of the *nuo*, *cyo*, and *sdh* operons to reduce cell membrane potential and induces tolerance to antisense PNA delivered by an arginine-rich peptide. (e) The Cpx represses the expression of the *nuo*, *cyo*, and *sdh* operons to inhibit the electron transport chain. (f) The Cpx ESR upregulates the expression of *marA* and *soxS*, inducing the expression of nitroreductases. The overexpression of nitroreductases promotes NIT prodrug activation and ROS accumulation. And the inhibition of the *nuo*, *cyo*, and *sdh* operons directly induces the ROS accumulation. (g) The Cpx ESR suppresses prodigiosin production by directly inhibiting the expression of the *pig* gene cluster. The Cpx ESR indirectly inhibits *xcnA-L* gene cluster but indirectly induces *xcnMN* gene cluster to reduce the production of Xcns and nematophin. (h) Glutamine inhibits CpxR indirectly through inosine, while BING inhibits CpxR directly. ϕ: electric potential; CPP-PNA: peptide nucleic acid (PNA) delivered by cell-penetrating peptides; ROS: reactive oxygen species; AMK: amikacin; NIT: nitrofurantoin; Xcns: xenocoumacins; IM: internal membrane; PP: periplasm; OM: outer membrane.

The effect of the Cpx response on aminoglycoside resistance had been long observed [79,80], and additional studies have revealed a link between the Cpx response and β-lactamases resistance [81,82]. With further research, the mechanism that the Cpx response regulates multidrug resistance has been extensively explained (Figure 3a-h). Since previous reviews have discussed the regulation of bacterial resistance through the Cpx response (see review [83]), this review only introduces recent developments and controversies in the field.

### Broad-spectrum antimicrobial resistance

The primary bactericidal mechanism of β-lactams involves damaging cell wall integrity by inhibiting peptidoglycan precursors biosynthesis, owing to their structural similarity to peptidoglycan precursors (see review [84]. Changes in cell wall structure can activate the Cpx response, as shown in various studies [85,86]. Additionally, one common mechanism of Gram-negative bacteria in developing antibiotic resistance is the reduction of cell permeability through the activation of Cpx response. Several studies have shown that the activation of the Cpx response regulates the expression of outer membrane porins (OmpF, OmpC, OmpD, and OmpW), reducing outer membrane permeability and enhancing tolerance to aminoglycosides, quinolones, and β-lactams [82,87–89] (Figure 3a). Furthermore, recent studies show that the Cpx response can induce the overexpression of the β-lactamase gene *ampC* to increase β-lactams resistance in *Klebsiella pneumoniae*. The cell wall modification gene *slt* belongs to the Cpx regulon, and upregulated in Cpx-inducing conditions [90]. Slt is responsible for the degradation of uncross-linked nascent PG into anhydro-disaccharide-pentapeptide when exposed to β-lactams [90,91]. This compound is converted to anhydro-monosaccharide-pentapeptide, which act as a signal for *ampC* induction [92,93]. Consequently, the resistance to β-lactam antibiotics could potentially be attributed to the up-regulation of *slt* mediated by the Cpx pathway [82]. However, the specific mechanism by which the Cpx response modifies the cell wall or OM to regulate drug resistance is not clear, even though the Cpx response can regulate the expression of peptidoglycan amidases AmiA and AmiC [94] (Figure 3c).

Actively pumping antibiotics out of cells via multidrug resistance (MDR) efflux pumps is another strategy commonly used by Gram-negative bacteria to increase drug resistance. Numerous studies have shown that the Cpx response confers bacterial resistance to β-lactamases, quinolones, aminoglycosides, and cationic antimicrobial peptides (cAPMs) by up-regulating MdtABCD, ArcAD-TolC, AcrAB-TolC, EmrKY, KpnEF, VexAB-VexGH, MexAB-OprM, MuxABC-OpmB efflux pumps [95–99] (Figure 3b). Moreover, the regulation of efflux pumps by the Cpx response and resulting resistance is observed in various bacteria and drugs, suggesting a universal mechanism of resistance regulation by the Cpx response through MDR efflux pumps across different bacteria.

### Aminoglycosides

Of course, aminoglycoside resistance is not solely due to increased drug efflux, as *tolC* deletion mutant strains still show resistance to these drugs [83]. In the model of aminoglycoside action, aminoglycosides must cross the plasma membrane in an energy-dependent way to cause protein mistranslation [87]. Therefore, it is easy to guess that the resistance of Cpx response to aminoglycosides may result from the effect on energy metabolism. Some current studies provide valuable insights into this hypothesis. The Cpx pathway inhibits the biosynthesis of succinate dehydrogenase (SDH) and the transcription of operons encoding NDH-I (*nuo*) and cytochrome *bo3* (*cyo*) [100–102] (Figure 3e). SDH is the only TCA cycle enzyme that directly connected to the electron transport chain (ETC), is the core of metabolism and energy conversion [103] (Figure 3e). And the function of the SDH complex is impaired in the Cpx deletion mutant strains [100]. The results show that the Cpx response is essential for maintaining energy levels and the stability, activity, and functionality of the ECT complex. Unfortunately, these studies only highlight the significant role of the Cpx response in regulating energy metabolism but do not clarify the connection between energy metabolism and aminoglycoside resistance.

Along with changes in energy metabolism, alterations of the proton motive force (PMF) in the ETC, aminoglycoside enter the cell in a PMF-dependent manner [104]. Previous studies have indicated that the Cpx mutations do not affect oxygen consumption or PMF [105]. However, recent studies have shown that oxygen consumption is also affected by the Cpx response [102]. And the Cpx enhances colistin’s antibacterial activity against *S. Typhimurium* by targeting ROS but does not impact PMF [106]. Further study, however, hints at another possibility: the Cpx response can alter PMF through respiration [107]. The plasma membrane PMF is composed of electric potential and the transmembrane proton gradient. When exposed to antisense peptide nucleic acid (PNA) delivered by an arginine-rich peptide, the constitutively activated Cpx response reduces membrane potential by decreasing respiration [107] (Figure 3d). Overall, further study is needed to understand the impact of the Cpx response on PMF.

Another proposed model suggests that the Cpx response upregulates protease expression to degrade mistranslated membrane proteins, enhancing resistance to aminoglycosides (see review [83]. Despite some data supporting both models, it is insufficient to clarify how the Cpx response regulates aminoglycoside resistance. Moreover, these models are not mutually exclusive. It is possible that the Cpx response increases aminoglycoside resistance both by altering respiration and degrading mistranslated proteins.

### ROS-mediated killing

Controversy surrounds whether antibiotics induce ROS through the Cpx response to cause cell death. In the ROS-mediated cell death model, bactericidal antibiotics activate the Cpx response. Crosslinking the Cpx pathway with the ArcAB TCS can induce cells to enter oxidative stress state, ultimately resulting in hydroxyl radical formation and cell death. This model was subsequently refuted, It was found that activating the Cpx response in wild-type *E. coli* increased resistance to aminoglycosides [81,108]. Additionally, the constitutively activated Cpx response in *E. coli* confers resistance to aminoglycoside antibiotics and hydroxyurea. It does not increase resistance to norfloxacin or ampicillin. Thus, the Cpx response confers resistance to partial antibiotics, suggesting that antibiotic may not kill bacteria via a common, ROS-related mechanism [109].

However, several recent studies have confirmed the important role of ROS induced by antimicrobial antibiotics through the Cpx response in mediating cell death. For example, Nie *et al*. demonstrated that the Cpx response in *S. typhimurium* can target ATP and ROS formation, enhancing the bactericidal effect of colistin [106]. Zhai *et al*. showed that a lethal dose of epigallocatechin gallate (EGCG) could activate the Cpx response, causing endogenous ROS accumulation, and ultimately resulting in *E. coli* cell death [110]. However, the strongest evidence supporting this model comes from the research findings of Ren *et al*. This study showed that the combination of amikacin (AMK) and nitrofurantoin (NIT) can significantly enhance ROS formation and efficiently eliminate uropathogens (Figure 3f). In this process, the Cpx-dependent overexpression of nitroreductases is the key to the synergistic effect [111] (Figure 3f). Increased nitroreductase activity increases ROS by activating NIT. Furthermore, increased nitroreductase activity decreases NADH and NADPH levels because these are cofactors for this enzyme. Therefore, the Cpx response increases ROS by upregulating nitroreductase expression, ultimately leading to cell death [111]. It is worth noting that aminoglycosides, in addition to AMK, can also synergize with NIT, likely due to their common bactericidal mechanism that causes protein misfolding. This misfolding can constitutively activate the Cpx response, forming the basis of this synergistic effect [111].

### Antimicrobial compounds production

The Cpx response can affect the production of bacteriocins and antimicrobial compounds, in addition to regulating resistance to exogenous antibiotics. In *Xenorhabdus nematophila*, the Cpx response negatively regulates *xcnA-L* but positively regulates the *xcn-MN* gene cluster expression, promoting nematophin while inhibiting Xenocoumacins synthesis [112,113] (Figure 3g). Even though the mechanism by which the Cpx response indirectly regulates *xcn* sites is still unclear, it also suggests the potential for modifying microbes in the industrial production of antimicrobial substances by manipulating the Cpx. Another study further contributes to this possibility. The Cpx response of *Serratia marcescens* is activated by inappropriate culture temperature, leading to the suppression of prodigiosin production by inhibiting precursors formation, energy genes related to its synthesis, and the transcription of its biosynthesis gene cluster *pig* [114] (Figure 3g). Therefore, eliminating the Cpx-mediated temperature sensing may be a measure to increase prodigiosin production in *S. marcescens*.

### Antimicrobial agents target

Based on the role of the Cpx response in antimicrobial resistance and ROS-mediated killing, it is evident that the Cpx ESR could be a valuable antibiotic target. Newly conducted studies also support this assertion, in addition to the cases mentioned earlier (colistin, EGCG, and synergistic effects of NIT and aminoglycosides). The novel AMP BING (Blocker of INter-membrane stress responses of Gram-negative bacteria) functions as a CpxR inhibitor, synergistically increasing the bactericidal effect of β-lactams like ampicillin, amoxycillin, and aminocoumarin, and delaying the emergence of antibiotic resistance at sublethal doses [115] (Figure 3h). Another broad-spectrum antibiotic adjuvant, glutamine, has a comparable impact by inhibiting the Cpx response through the promotion of nucleoside biosynthesis, such as inosine, to eliminate multidrug-resistant uropathogens [116] (Figure 3h). Furthermore, glutamine also hampers the progression of ampicillin resistance [116].

## Inter-kingdom signaling

The communication between microorganisms and their hosts is dubbed inter-kingdom signaling [117]. Cell-surface receptors play a crucial role in this process [117]. A common signaling receptor in bacteria is QseBC, which senses the autoinducer 2 and 3, enabling communication between the microbe and its host [118,119]. However, recent studies have shown that the Cpx response can also serve as a cell envelope receptor involved in inter-kingdom signaling [120–122].

### Norepinephrine

When exposed to host norepinephrine (NE), *Salmonella typhi* promotes the invasion of host cells by secreting hemolysin. This NE-mediated hemolytic phenotype is regulated by the CpxAR TCS and is not dependent on the established *E. coli*_O157:H7_ adrenergic receptor QseBC [120]. Thus, the Cpx ESR could be described as a novel adrenergic receptor involved in inter-kingdom signaling [5,120].

### Serotonin and indole

**Figure 4.**
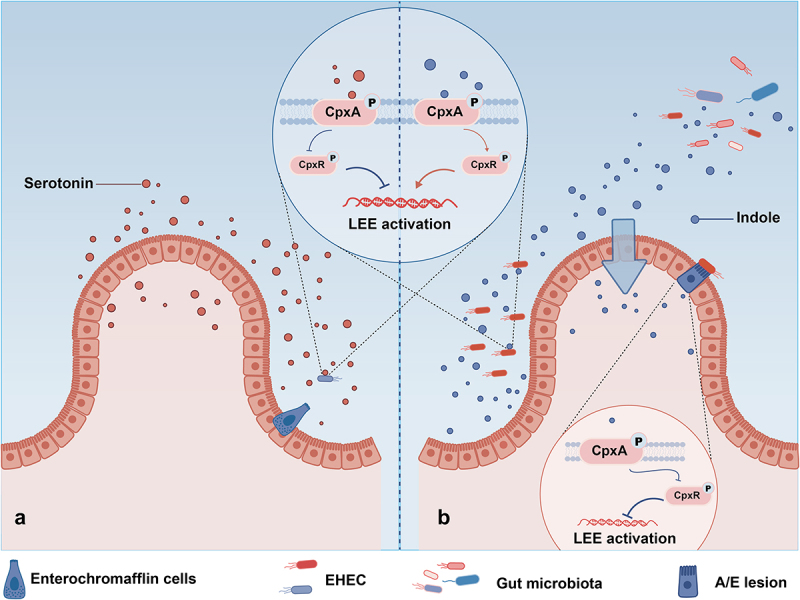
The Cpx ESR regulates interkingdom signaling in the gut. (a) High levels of serotonin in the lumen inhibit CpxA phosphorylation, thereby inhibiting LEE locus activation. (b) Indole is secreted into the lumen by the microbiota and then absorbed by the intestinal epithelium. The high concentration of indole in the lumen activates CpxA phosphorylation, thereby activating the downstream LEE locus. The lower concentration of indole in the intestinal epithelium inhibits CpxA and thus inhibits LEE locus activation.

As a bacterial serotonin receptor, CpxA detects serotonin levels from the host, and transmits this signal to pathogens, regulating the expression of virulence factors [121]. Serotonin is synthesized by enterochromaffin cells, the specialized intestinal epithelial cells responsible for producing about 90% of the body’s serotonin [123–125]. The synthesized serotonin is and then released into the lamina propria and lumen. Serotonin signaling in the intestinal mucosa is terminated by the serotonin selective reuptake transporter [126]. At high serotonin concentrations, CpxA is dephosphorylated by serotonin, blocking the transcription of the LEE locus. This reduces the virulence of intestinal pathogens like enterohemorrhagic *E. coli* (EHEC) and *C. rodentium*, thereby decreasing the host’s susceptibility to these pathogens [121] (Figure 4a).

In addition to the serotonin secreted by the host, CpxA also senses indole produced by microbiome metabolism (Figure 4b). Exogenous indole is synthesized by the microbiota and secrets into the lumen, where the indole is absorbed by intestinal epithelial cells to improve their barrier function [127]. The CpxA in intestinal pathogens like EHEC and *C. rodentium* can sense exogenous indole levels. It down-regulates the transcription of the LEE locus at low concentrations and up-regulates the expression at high concentrations [122] (Figure 4b). The concentration of exogenous indole is higher in the lumen than in the intestinal epithelium [122]. By sensing the gradient of exogenous indole concentration, CpxA ensures that intestinal pathogens like EHEC and *C. rodentium* express virulence genes only in the intestinal epithelium, which is the colonization niche. Therefore, CpxA, as an indole sensor, mediates the host-microbiota-pathogen signaling.

It is important to highlight that slight alterations in the chemical structure of serotonin do not diminish its impact on LEE gene expression. This observation aligns with the result that CpxA senses indole due to the structural similarities shared between serotonin and indole, both of which are derived from tryptophan [121]. CpxA senses chemical signals from both the bacteria and the host, showing biochemically convergent in inter-kingdom chemical signaling. Sensing signal integration between host and bacteria through an HK receptor seems a common signaling mechanism in the biological kingdom, with CpxA and QseC both having analogous functions [117,121].

## Conclusion

It is unrealistic to depend solely on the Cpx response to regulate all the physiological functions mentioned above; other systems are also required to provide assistance. For instance, the Cpx ESR interacts with the EnvZ/OmpR system through the positive regulation of MzrA, contributing to the cellular response to hyperosmotic stress. The Cpx pathway involves in the upregulation of ArcD and MdtA efflux pumps by amplifying the function of BaeSR system. Moreover, interactions between Cpx and σ^E^, σ^S^, and small non-coding RNA (sRNA) have been documented in literatures (see review [128]. The Cpx ESR not only establishes connections by regulating signaling proteins in other regulatory pathways, but its activity is also affected by other stress responses. These regulatory pathways are interconnected to form an extensive network that regulates the interaction between pathogens and hosts.

The mechanisms of the Cpx response in monitoring and responding to envelope stress have been studied for many years. We now know that the Cpx response senses and responds to changes in the external environment, regulates the expression of virulence factors based on their relative location to the host, and mitigates the bactericidal effects of antimicrobials. Based on the role of the Cpx response in responding to antimicrobials and regulating bacterial resistance, many antibacterial agents or antibiotic combinations targeted at the Cpx ESR have achieved positive outcomes.

The Cpx ESR still mediates host-pathogen inter-kingdom signaling, although it is only found in intestinal pathogens. Moreover, the Cpx response has the capacity to sense external stimuli and regulate virulence factor expression and antimicrobial resistance. All these supports the crucial role of the Cpx ESR in regulating the intricate network of interactions between pathogens and hosts. Nonetheless, the molecular mechanism by which the Cpx ESR regulates all these physiological functions remains unknown. Further elucidation of these detailed mechanisms is expected to enhance our ability to eradicate bacteria in healthcare and industrial production in the future.

## Data Availability

Data sharing is not applicable to this article as no new data were created or analyzed in this study.
